# Antiviral Function of NKEF against VHSV in Rainbow Trout

**DOI:** 10.3390/biology10101045

**Published:** 2021-10-15

**Authors:** Veronica Chico, Maria Elizabhet Salvador-Mira, Ivan Nombela, Sara Puente-Marin, Luis Perez, Luis Mercado, Maria del Mar Ortega-Villaizan

**Affiliations:** 1 Instituto de Biología Molecular y Celular (IBMC), Universidad Miguel Hernández (IBMC-UMH), 03202 Elche, Spain; vchico@umh.es (V.C.); maria.salvador04@goumh.umh.es (M.E.S.-M.); ivan.nombela@kuleuven.be (I.N.); sara.puentes@liu.se (S.P.-M.); luis.perez@umh.es (L.P.); 2 Instituto de Investigación, Desarrollo e Innovación en Biotecnología Sanitaria de Elche (IDiBE), Universidad Miguel Hernández (IDiBE-UMH), 03202 Elche, Spain; 3 Instituto de Biología, Pontificia Universidad Católica de Valparaíso, Valparaíso 2373223, Chile; luis.mercado@pucv.cl

**Keywords:** erythrocyte, red blood cells, NKEF, peroxiredoxin, rainbow trout, VHSV, antiviral, therapeutic

## Abstract

**Simple Summary:**

An antioxidant protein has been identified in a sample of erythrocytes exposed to a fish virus. We evaluated the role of this protein as an antiviral molecule in fish. Through silencing and overexpression assays we determined the antiviral effect of this protein in the infectivity of the virus. In conclusion, this antioxidant protein may be a potential target for new therapeutic strategies against viral infections.

**Abstract:**

Natural killer enhancing factor (NKEF) belongs to the peroxiredoxin family of proteins, a group of antioxidants that has been extensively studied in mammals. Recently, we identified NKEF in the immunoprecipitated proteome of rainbow trout red blood cells (RBCs) exposed to viral hemorrhagic septicemia virus (VHSV). In the present study, we evaluated the role of NKEF in the antiviral response of rainbow trout against VHSV by examining the expression profile of NKEF in VHSV-exposed RBCs and rainbow trout gonad-2 (RTG-2) cell line. We found an in vitro correlation between decreased VHSV replication and increased NKEF expression after RBCs were exposed to VHSV, however this was not found in RTG-2 cells where the infection highly increased and *nkef* transcripts remained almost unchanged. In addition, siRNA silencing of the *nkef* gene in rainbow trout RBCs and RTG-2 cells resulted in increased VHSV replication. We also found a correlation between *nkef* gene silencing and a decrease in the expression of genes related to type 1 interferon (IFN1) pathway. These findings indicated that NKEF is involved in the antiviral mechanisms of rainbow trout RBCs against VHSV and thus support its antiviral role and implication in the modulation of their immune response. Finally, overexpression of NKEF in an EPC cell line significantly reduced VHSV infectivity and was coupled to an increment in IFN1-related genes. In conclusion, NKEF may be a potential target for new therapeutic strategies against viral infections.

## 1. Introduction

The peroxiredoxin (PRDX) family is a highly conserved gene family, containing six members that are expressed in a broad range of organisms, including both prokaryotes and eukaryotes [1]. PRDX, first isolated and purified from *Saccharomyces cerevisiae*, was initially described as a soluble protein that specifically inhibits the inactivation of diverse enzymes by a nonenzymatic Fe^3+^/O2/thiol mixed-function oxidase system [2]. In humans, PRDX was first identified in red blood cells (RBCs) and was named as natural killer enhancing factor (NKEF) due to its ability to increase the cytotoxicity of NK cells against tumor cells [3]. Human NKEF is a 44-kDa protein consisting of two subunits: NKEF-A and -B, identified as PRDX1 and PRDX2, respectively [4]. Apart from its antioxidant function, NKEF contributes to an extensive array of cellular functions, such as apoptosis modulation [5,6], cell proliferation and differentiation [7,8], modulation of inflammation in pathogen infection and tissue damage [9,10], and antiviral activity [11]. 

NKEF has been characterized in many fish species, including rainbow trout [12]. Recently, several studies looked at the role of NKEF in the fish immune response as well as NKEF gene and protein expression levels after bacterial, viral, and parasitic infections [13]. In teleost viral infections, *nkef* transcripts expression increased in the peripheral blood leukocytes, head kidney, and blood of rainbow trout challenged with viral hemorrhagic septicemia virus (VHSV) [14,15] and in the peripheral blood leukocytes of common carp challenged with spring viremia carp virus (SVCV) [16]. Moreover, *nkef* transcript levels increased in the rainbow trout monocyte-macrophage cell line RTS11 [17] and in VHSV-exposed RBCs [18], as well as in nodavirus-infected seabream and sea bass brain and head kidney [19]. In addition, we identified the NKEF protein in the immunoprecipitated proteome of VHSV-exposed rainbow trout RBCs by using an antibody against the N protein of VHSV [20]. Several mechanisms have been proposed for the antiviral response against VHSV in rainbow trout RBCs [18,20,21,22], but the role of NKEF in this context is not known yet. 

Based on this prior knowledge, we evaluated the role of NKEF in the antiviral response of rainbow trout against VHSV using RBCs and RTG-2 as cell models. In summary, we demonstrated the antiviral effect of NKEF in the infectivity of VHSV through protein silencing and overexpression in different fish cell types. Moreover, we demonstrated the implication of NKEF in the modulation of the IFN1-related cell response. All these results suggested that NKEF may be a potential target for new therapeutic strategies against viral infections.

## 2. Materials and Methods

### 2.1. Animals and Ethical Statements

Rainbow trout (Oncorhynchus mykiss) individuals of approximately 6–7 cm in size were acquired from Piszolla S.L., Cimballa Fish Farm (Zaragoza, Spain) and maintained at the University Miguel Hernández (UMH) animal house facilities. They were maintained at 14 °C with dechlorinated water in a recirculating water system. Animals were adapted to laboratory conditions for 2 weeks previous to experimentation. 

### 2.2. Cell Culture and Virus

The protocol used to obtain and purify rainbow trout RBCs has been previously described [18,23]. In summary, rainbow trout blood from the caudal vein was extracted using insulin syringes (NIPRO, Bridgewater, NJ, USA). Then, the purification of RBCs was carried out by two steps of Ficoll density gradient centrifugations (7206 g, Ficoll 1.007; Sigma-Aldrich, Madrid, Spain). After that, Ficoll-purified RBCs were cultured in 25 cm^2^ flasks (Nunc, Roskilde, Denmark)with RPMI-1640 medium (Dutch modification) (Gibco, Thermo Fisher Scientific, Carlsbad, CA, USA) containing 10% fetal bovine serum (FBS) gamma irradiated (Cultek, Madrid, Spain), 2 mM L-glutamine (Gibco),1 mM pyruvate (Gibco), 2 μg/mL fungizone (Gibco), 50 μg/mL gentamicin (Gibco), 100 U/mL penicillin (Sigma-Aldrich), and 100 μg/mL streptomycin (Sigma-Aldrich) at 14 °C for 24 h.

The fish cell line rainbow trout gonad-2 (RTG-2) was purchased from the American Type Culture Collection (ATCC) (ATCC CCL-55) and maintained in MEM medium (Sigma-Aldrich) supplemented with 10% FBS, 2 mM L-glutamine, 1 mM pyruvate, 2 μg/mL fungizone, and 50 μg/mL gentamicin at 21 °C.

Another fish cell line used was the fathead minnow Epithelioma papulosum cyprini (EPC) cell line, acquired from ATCC (ATCC CRL-2872) and maintained in RPMI-1640 medium (Dutch modification) (Gibco, Thermo Fisher Scientific) with 10% FBS, 2 mM L-glutamine, 1 mM pyruvate, 2 μg/mL fungizone, and 50 μg/mL gentamicin at 28 °C.

The fish virus VHSV, strain 07.71 [24] (ATCC VR-1388), was produced in EPC cells at 14 °C as previously explained [25].

### 2.3. Time Course of NKEF Expression in VHSV-Exposed RBCs and RTG-2 Cells

Ficoll-purified RBCs (10^6^) were exposed to VHSV at a multiplicity of infection (MOI) of 1 in RPMI 2% FBS medium at 14 °C. After 3 h, the medium was refreshed with RPMI 2% FBS. The transcript and protein expression levels of NKEF were evaluated at different time points postexposure (0, 3, 6, 24, and 72 hours postexposure (hpe)). To analyze *nkef* transcript levels, cells were added TRK lysis buffer (Omega Bio-Tek, Inc., Norcross, GA, USA) and kept at −80 °C as previously described [18]. Then, *nkef* transcript expression level was evaluated by real-time RT-qPCR. To normalize the data, every time point in the graph was relativized to its respective control (nonexposed to VHSV). To analyze NKEF protein levels at 6 and 24 hpe, by flow cytometry, cells were fixed, permeabilized, and incubated with the primary and secondary antibodies indicated below and as previously described [18].

It is necessary to highlight that we used RTG-2 cells (2.5 × 10^5^) since it is a cell line that is susceptible to VHSV infection (in contrast to RBCs). For that reason, these cells were infected with VHSV at a MOI of 0.1 in MEM 2% FBS medium at 14 °C. After 1.5 h, the medium was refreshed with MEM 2% FBS. The transcript expression level of *nkef* was evaluated at different time points post-exposure (0, 3, 6, 24, and 72 hours postexposure (hpe)) by RT-qPCR, as described below.

### 2.4. VHSV Challenged of Rainbow Trout

Rainbow trout (6 to 7cm in size) were intramuscularly injected with 50 µL of RPMI 2% FBS medium with VHSV (10^8^ tissue culture infectious dose 50% (TCID50)/mL). Mock-infected fish were injected with 50 µL of sterile RPMI 2% FBS and used as a control. At 48 h postchallenge, fish were euthanized with ethyl 3-aminobenzoate methanesulfonate (MS-222) (Sigma-Aldrich) and different tissues were extracted (spleen, head kidney and blood). RBCs were purified by single-cell sorting using the BD FACS Jazz cell sorter (BD Biosciences, Madrid, Spain) as previously described [21]. Tissue samples were stored in TRK lysis buffer for RNA extraction and stored at −80 °C [21].

### 2.5. NKEF siRNA Assay

Two NKEF small interfering RNA sequences (siNKEF) were designed and synthesized by Sigma-Aldrich (Table 1) to perform NKEF silencing.

To evaluate NKEF gene silencing at gene level, RBCs and RTG-2 cells were electroporated with a mixture of 2 siNKEF sequences using the Neon Transfection system (Life Technologies, Thermo Fisher Scientific, Carlsbad, CA, USA). In the case of RBCs, electroporation was carried out with 187 pmol of each siRNA per 0.5 × 10^6^ cells resuspended in Buffer T (Life Technologies). For RTG-2, electroporation was performed with 68 pmol of each siRNA per 1.23 × 10^5^ cells resuspended in Buffer R (Life Technologies). siGFP (Sigma-Aldrich) was used as negative control (187 pmol for RBCs and 68 pmol for RTG-2 cells). Then cells were cultured for 24 h. After that time, the cell pellet was stored in TRK lysis buffer for RNA extraction and stored at −80 °C.

NKEF gene silencing was evaluated at the protein level in RTG-2 cells by Western blot as previously described [18]. Briefly, RTG-2 cells were electroporated with 100 pmol of each siRNA per 1.23 × 10^5^ cells resuspended in Buffer R (Life Technologies) with 100 pmol of siGFP (Sigma-Aldrich) as a negative control. After the time of incubation of 24 and 48 h, cells were collected, homogenized, and loaded on Tris-Glycine sodium dodecyl sulfate (SDS) (NZYTech, Lisboa, Portugal) 16% polyacrylamide (NZYTech) gel under reducing conditions. Then, the gel was transferred to a 0.4-µm pore nitrocellulose membrane (BioRad, Madrid, Spain) and blocked with 8% dry milk and 1% Tween-20 (Merck, Madrid, Spain) in PBS. The membrane was incubated with a mouse anti-NKEF antibody (1/25) provided by Dr. Luis Mercado [26] as a primary antibody. Anti-α-actin antibody (Sigma-Aldrich, Ref. A2066) (1/100) was used as an endogenous control. Rabbit antimouse peroxidase and goat antirabbit peroxidase (Sigma-Aldrich) were used as secondary antibodies for anti-NKEF and anti-α-actin, respectively. Proteins were detected with ECL chemiluminescence reagents (Amersham Biosciences, Buckinghamshire, UK) and revealed using the ChemiDoc Imaging System (BioRad). For the endogenous protein α-actin, the same membrane was stripped with stripping buffer (10%SDS (NZYTech), 1M Tris HCl (Sigma-Aldrich) pH 6.8, 100 mM β—Mercaptoetanol (Sigma-Aldrich)) at 56 °C, for 45 min, washed three times with ddH_2_O, blocked as described above, and incubated with primary and secondary antibodies. Band densitometry was analyzed by selecting the bands with the rectangle tool, using Scion Image software (Scion Corp., Frederick, MD, USA)

### 2.6. Evaluation of VHSV Infectivity in Cells Treated with siNKEF

To evaluate the role of NKEF in the response of RBCs to VHSV, RBCs were transfected with siNKEF or siGFP (187 pmol of siNKEF or siGFP per 0.5 × 10^6^ cells) resuspended in Buffer T (Life Technologies). At 24 h post-transfection, RBCs were exposed to VHSV MOI 1 at 14 °C. In the case of RTG-2 cells, to evaluate the role of NKEF in the response to VHSV, they were transfected with siNKEF or siGFP (100 pmol of siNKEF or siGFP per 1.23 × 10^5^ cells) resuspended in Buffer R (Life Technologies). At 48 h post-transfection, cells were exposed to VHSV MOI 0.1 (RTG-2) at 14 °C. In both cases, at 3 hpe, medium was refreshed with RPMI 2% FBS and incubated for 24 h. Then, cells were resuspended in TRK lysis buffer for RNA extraction and kept at −80 °C.

### 2.7. RNA Isolation, RT-PCR, and RT-qPCR

E.Z.N.A. Total RNA Kit (Omega Bio-Tek) was used for RNA extraction as previously described [18]. Genomic DNA was eliminated from RNA samples using the TURBO DNAse kit (Ambion, Thermo Fisher Scientific) as described previously [18]. Subsequent cDNA synthesis was carried out using M-MLV reverse transcriptase (Invitrogen, Thermo Fisher Scientific) as described previously [27].

Semiquantitative RT-PCR was performed using GeneAmp PCR System 2700 thermocycler (Applied Biosystems, Thermo Fisher Scientific) as described previously [20]. Primers used to perform RT-PCR are shown in Table 2. For normalization, glyceraldehyde 3-phosphate dehydrogenase (*gapdh*) and β-actin genes were utilized as endogenous controls. PCR products were identified on a 1.2% agarose gel stained with Gelred nucleic acid stain (Biotium, Inc., Fremont, CA, USA).

Real-time RT-qPCR was carried out using QUANTSTUDIO 3 system (Applied Biosystems, Thermo Fisher Scientific Inc.). Cycling conditions and gene expression analysis methods were performed as described previously [18]. Primers and probes are included in Table 3. The endogenous control used was elongation factor 1 α (*ef1α*).

### 2.8. Flow Cytometry

The protocol used to fix, permeabilize, and stain cells with primary and secondary antibodies is described elsewhere [18]. The primary antibody was mouse anti-NKEF [26] (provided by Dr. Luis Mercado) at a 1/50 dilution in permeabilization buffer. Antimouse IgG 488 (Sigma-Aldrich) produced in goat was used as secondary antibody, at a 1/200 dilution. Samples were analyzed using FACSCanto II (BD Biosciences, Madrid, Spain) flow cytometer. A total of 30,000 events were acquired.

### 2.9. nkef cDNA Cloning in pmTFP Plasmid

The *nkef* cDNA sequence (accession number: AF250194.1) was cloned into pmTFP1 (Allele Biotechnology, ABP-FP-TCNCS), which encodes the teal fluorescent protein 1 (TFP1) [34] at ShineGene Bio-Technologies, Inc. (Shanghai, China). pmTFP1-NKEF was used in the transfection assays as described here.

For plasmid production, the competent Escherichia coli strain XL1-blue was transformed with pmTFP1-NKEF by thermal shock. Bacteria were cultured on LB agar plates with ampicillin (Sigma-Aldrich) and incubated overnight at 37 °C. Colonies obtained on agar plates were cultured in 200 mL of LB supplemented with ampicillin and incubated on an orbital shaker at 250 rpm at 37 °C overnight. Plasmid purification was performed using the QIAGEN Plasmid Midi kit (QIAGEN Inc., Madrid, Spain).

### 2.10. Transfection Assays of RBCs and EPC Cells with pmTFP1-NKEF

Cell transfection assays were performed by electroporation using the Neon Transfection System. Cells were electroporated with pmTFP1-NKEF at different plasmid concentrations (for RBCs: 0.5 μg, 1 μg, and 2 μg; for EPC: 50 ng, 100 ng, and 150 ng) or with pmTFP1 (RBCs: 2 µg and EPC: 250 ng) at 1600 V, 30 ms, 1 pulse. EPC cells were chosen for this assay because they are highly susceptible to gene transfection [35]. The protein expression of NKEF in transfected cells was monitored at different time points (for RBCs: 1, 3, and 6 days post-transfection; for EPC: 1, 2, and 3 days post-transfection) by evaluating the fluorescence emitted by the reporter protein TFP1 bound to NKEF protein (NKEF-mTFP1) using the IN Cell Analyzer 6000 microscope (GE Healthcare, Little Chalfont, UK) and quantified by flow cytometry using the FACSCanto II flow cytometer (BD Biosciences).

Moreover, cells were resuspended in TRK lysis buffer for RNA extraction to evaluate the immune response triggered by NKEF overexpression by means of RT-qPCR.

### 2.11. VHSV Infectivity in pmTFP1-NKEF-Transfected EPC Cells

To determine the protection conferred against VHSV by NKEF overexpression, we used EPC cells since they are easily transfectable, they are susceptible to VHSV infection (in contrast to RBCs), and do not endogenously express the rainbow trout NKEF. For that, EPC cells were transfected with pmTFP1-NKEF (150 ng) or pmTFP1 (250 ng) as a control. At 48 h post-transfection, the cells were infected with VHSV at MOI 0.1 in RPMI 2% FBS for 1 h at 14 °C. The medium was then refreshed and cells were incubated for 24 h at 14 °C. Supernatant was collected and kept at −80 °C for virus titration by focus forming units (FFU) assay. The cell pellet was resuspended in RNA extraction buffer to calculate viral replication and immune response by means of RT-qPCR using primers shown in Table 3.

FFU/mL in the supernatant was calculated as previously described [23]. Briefly, EPC cells were incubated with supernatant serial dilutions (10^−1^, 10^−2^, 10^−3^) in RPMI 2% FBS. After 2 h at 14 °C, the cell medium was refreshed and cells were incubated for an additional 24 h. Then, cells were fixed with paraformaldehyde (PFA) (Sigma-Aldrich) diluted at 4% in PBS followed by a second fixation with cold methanol (Sigma-Aldrich) and permeabilized with 0.3% Triton X-100 (Sigma-Aldrich). Monoclonal murine 2C9 antibody against N protein of VHSV (at 1/3000 dilution) was used as primary antibody [36] and antimouse IgG 488 (Sigma-Aldrich) (at 1/300 dilution) was used as a secondary antibody. Immunofluorescence images were taken using the IN Cell Analyzer 6000 imaging system (GE Healthcare). FFU/mL were counted manually and are represented as viral yield (percentage to positive control FFU/mL).

### 2.12. Software and Statistics

The statistics calculations and graphic representations were carried out with Graphpad Prism 6 software (San Diego, CA, USA). The statistic tests and associated *p*-values are indicated at each assay. Flowing Software 2.5.1 (www.flowingsoftware.com/ accessed on 14 September 2021) was used to process and analyze flow cytometry data.

## 3. Results

### 3.1. Time Course of NKEF Expression in RBCs Exposed to VHSV

**Figure 1:**
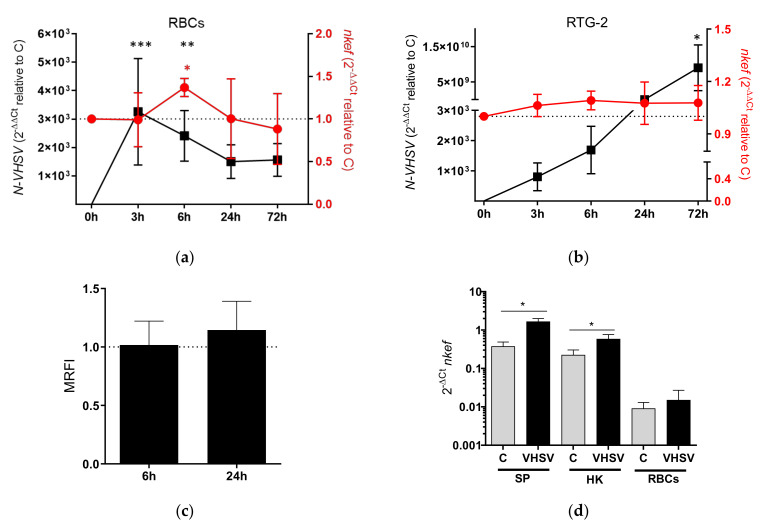
NKEF expression is increased in rainbow trout RBCs after VHSV exposure. Time course of *nkef* gene expression in (**a**) RBCs and (**b**) RTG-2 cells exposed to VHSV at a multiplicity of infection (MOI) of 1 at different time points post-exposure. The graph shows the RT-qPCR gene expression profiles for *nkef* in red and the N-VHSV gene in black. Gene expression was normalized to control cells (C, unexposed to virus), dashed line. The endogenous gene control used was *om-ef1α.* h indicates hours. Data represent the mean ± SD of n = 6 individuals for RBCs and n = 3 experiments in RTG-2. For statistical analysis, the Kruskal–Wallis test and Dunn’s multiple comparison post hoc test were performed. *, **, and *** indicate *p*-values < 0.05, <0.01 and <0.001, respectively, for the N-VHSV gene (black asterisks) and for *nkef* (red asterisk), and normalized to control cells (unexposed to virus) at each respective time point. (**c**) NKEF protein expression in VHSV-exposed RBCs at 6 and 24 hpe analyzed by flow cytometry. Mean relative fluorescent intensity (MRFI), relative to control cells (dashed line) is represented in the graph. h indicates hours. Data show the mean ± SD of n = 7 individuals. The Kruskal–Wallis test and Dunn’s multiple comparison post hoc tests were performed and compared with the control. (**d**) *nkef* gene expression in different tissues (spleen [SP], head kidney [HK] and RBCs) from rainbow trout challenged with VHSV at 48 h post-challenge. RT-qPCR was used to analyze data. *Om-ef1α* was used as reference gene. C means mock-infected rainbow trout. Data represent the mean ± SD of n = 2 individuals. Mann–Whitney analysis was used for statistical analysis. * indicates *p*-value < 0.05.

We determined the expression profile of the *nkef* gene by RT-qPCR in RBCs after VHSV exposure for 0, 3, 6, 24, and 72 h. We evaluated VHSV replication in RBCs by N-VHSV gene expression at each time point. *nkef* transcripts expression reached the highest expression level at 6 hpe, coinciding with the decline of VHSV replication (Figure 1a). At 6 hpe, *nkef* transcripts expression decreased until reaching basal levels at 72 hpe. However, in RTG-2 cells, a cell line that is susceptible to VHSV infection (in contrast to RBCs), *nkef* transcript levels slightly increased at 3 hpe (Figure 1b). In contrast to findings in RBCs, VHSV replication in RTG-2 cells continued until the cell monolayer was destroyed at 72 hpe.

We evaluated the protein expression of NKEF in VHSV-exposed RBCs by flow cytometry (Figure 1c) using a specific antibody against NKEF. The highest NKEF protein expression was observed at 24 hpe, in contrast to the highest *nkef* transcripts expression observed at 6 hpe.

In addition, we analyzed *nkef* gene expression in different tissues from rainbow trout mock-infected or challenged with VHSV (Figure 1d). The *nkef* transcripts were up-regulated in all the tissues analyzed from VHSV-challenged rainbow trout samples, being spleen where the highest expression level was found.

### 3.2. nkef Gene Silencing in RBCs and RTG-2 Cell Line

**Figure 2:**
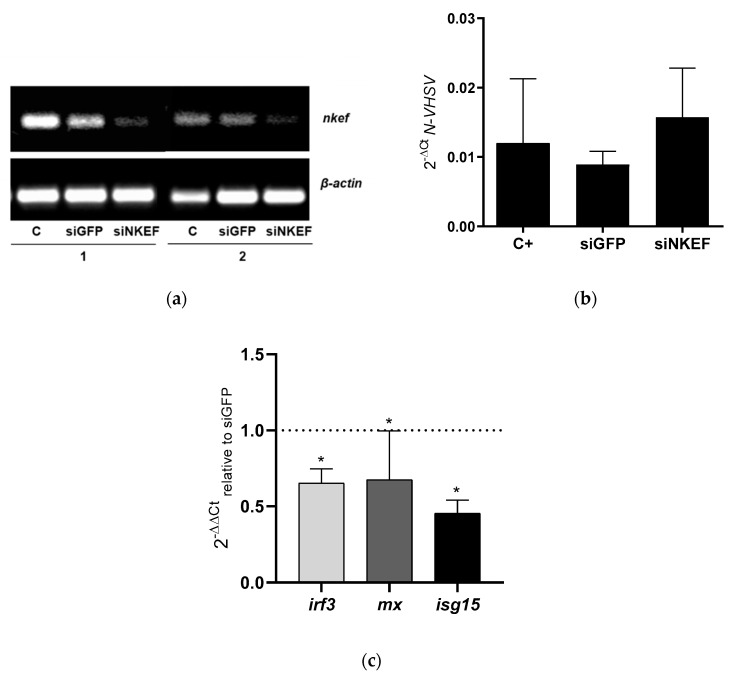
The effect of NKEF gene silencing on VHSV replication in RBCs. (**a**) RBCs were electroporated with siNKEF sequences. *nkef* gene expression was evaluated by RT-PCR after 24 h. siGFP was used as a negative control, and *β-actin* was used as endogenous control gene. C indicates control, and 1 and 2 indicate the sample number. (**b**) RBCs were transfected with siNKEF and after 24 h they were exposed to VHSV MOI 1 for 24 h at 14 °C. VHSV replication was evaluated by N-VHSV gene expression by RT-qPCR. *Om-ef1α* was used as the endogenous control gene. C+ means RBCs mock electroporated and exposed to VHSV. Data represent the mean ± SD of three individuals. The Kruskal–Wallis test and Dunn’s multiple comparison post hoc test were performed. (**c**) Evaluation of IFN1-related genes modulation by RT-qPCR in RBCs transfected with siNKEF and exposed to VHSV. Bars represented the expression of *irf3* (light grey), *mx* (dark grey) and *isg15* (black). Gene expression was normalized to RBCs transfected with siGFP and exposed to VHSV, dashed line. The endogenous control gene used was *om-ef1α* Data represent the mean ± SD of n = 4 individuals. The Mann–Whitney test was performed for statistical analysis between siGFP and siNKEF transfected RBCs and exposed to VHSV. * indicates *p*-value < 0.05.

We analyzed the implication of NKEF in VHSV replication using *nkef* gene silencing with siRNA. We detected a decrease in *nkef* gene expression, by means of RT-PCR, at 24 h post-transfection in RBCs transfected with siNKEF (Figure 2a). At protein level, unfortunately, we were not able to detect the NKEF protein since the RBCs’ hemoglobin interfered with NKEF band in the Western blot (data not shown). At functional level, RBCs transfected with siNKEF and exposed to VHSV, showed an increment in VHSV replication at 24 hpe (Figure 2b), just the opposite result observed in Figure 1a where NKEF is not silenced.

**Figure 3:**
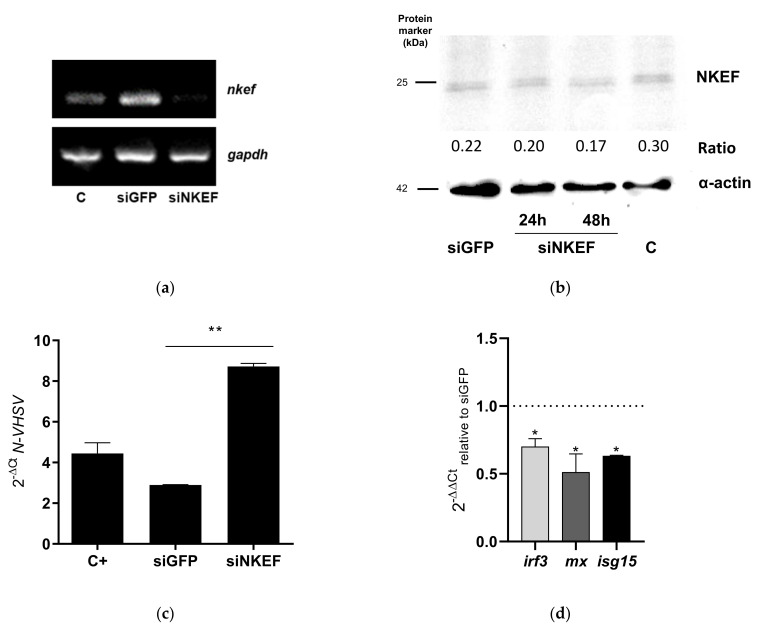
The effect of NKEF gene silencing on VHSV replication in RTG-2. (**a**) RTG-2 cells were electroporated with siNKEF sequences. *nkef* gene expression was evaluated by RT-PCR after 24 h. siGFP was used as a negative control, and *gapdh* was used as endogenous control genes. (**b**) Representative image of Western blot of NKEF protein levels after NKEF gene silencing in RTG-2 cells after 24 and 48 h using anti-NKEF antibody. siGFP was used as negative control. C indicates electroporated control cells, and h means hours. The endogenous protein control was α-actin, detected using anti-α-actin antibody. Ratio represents expression of NKEF relative to α-actin expression. (**c**) RTG-2 cells were transfected with siNKEF and after 48 h they were exposed to VHSV MOI 0.1 for 24 h at 14 °C. siGFP was used as negative control. VHSV replication was evaluated by N-VHSV gene expression by RT-qPCR. N-VHSV gene expression. *Om-ef1α* was used as the endogenous control gene. C+ means RTG-2 mock electroporated and exposed to VHSV. Data represent the mean ± SD of two experiments. The Kruskal–Wallis test and Dunn’s multiple comparison post hoc test were performed. ** indicates *p*-value < 0.01. (**d**) Analysis of IFN1 pathway modulation by RT-qPCR of *irf3*, *mx* and *isg15* genes in RTG-2 silenced with siNKEF and exposed to VHSV. Bars represent the expression of *irf3* (light grey), *mx* (dark grey) and *isg15* (black). Gene expression was normalized to cells transfected with siGFP and infected with VHSV, dashed line. The endogenous gene control used was *om-ef1α.* Data represent the mean ± SD of 2 experiments. A Mann–Whitney test was performed for statistical analysis between siGFP and siNKEF transfected cells and infected with VHSV. * indicates *p*-value < 0.05.

Similar results were obtained in RTG-2 cells transfected with siNKEF, where we detected a decrease in *nkef* gene and protein expression in RTG-2 cells transfected with siNKEF (Figure 3a,b). After 48 h, the amount of NKEF protein decreased relative to siGFP or mock electroporated control (Figure 3b and Supplementary Figure S1). Similarly, an increment in VHSV replication was observed in RTG-2 cells transfected with siNKEF (Figure 3c).

In an attempt to investigate the implication of NKEF in the antiviral immune response in RBCs and RTG-2 cells, we evaluated the expression level of genes related to IFN1 pathway after *nkef* gene silencing and incubation with VHSV. The results showed that the expression level of IFN1-related genes such as interferon regulatory factor 3 (*irf3*), Mx dynamin like GTPase (*mx*), and interferon-stimulated gene15 (*isg15*) were significantly decreased in RBCs and RTG-2 cells transfected with siNKEF and exposed to VHSV (Figure 2c and Figure 3d).

### 3.3. Overexpression of NKEF in RBCs

**Figure 4:**
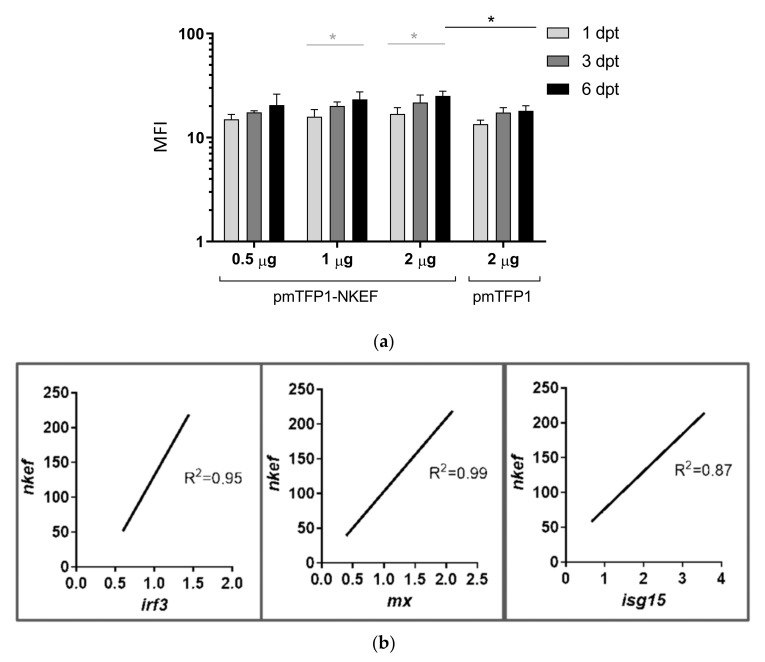
Time course of RBCs transfected with pmTFP1-NKEF. RBCs were electroporated with the plasmid pmTFP1-NKEF at different concentrations (0.5 µg, 1 µg, and 2 µg). NKEF expression was monitored at various time points. (**a**) Evaluation of NKEF protein levels using flow cytometry via the fluorescence emitted by the NKEF-bound TFP1 protein. Data represent mean fluorescent intensity (MFI)(mean ± SD of n = 3 individuals). A two-way ANOVA and Tukey’s multiple comparisons test were performed for statistical analysis, between plasmid concentrations (black lines and asterisks) and times post-transfection (gray lines and asterisks). * indicates *p*-value < 0.05. (**b**) Analysis of the IFN1-related genes *irf3*, *mx*, and *isg15* modulation in RBCs transfected with the plasmid pmTFP1-NKEF. Gene expression was evaluated by RT-qPCR. Linear regression was calculated between *nkef* gene expression and interferon stimulated genes *irf3*, *mx*, and *isg15* in pmTFP1-NKEF transfected RBCs (with 2 µg of plasmid and 3 dpt). Gene expression was normalized to pmTFP1 transfected RBCs. The endogenous gene control used was *om-ef1α.* Data are displayed as a linear regression line (R^2^: coefficient of correlation), n = 3 individuals.

NKEF expression in RBCs transfected with pmTFP1-NKEF was evaluated at different concentrations (0.5, 1, and 2 µg) and time points (1, 3, and 6 days post-transfection) by flow cytometry. In RBCs transfected with pmTFP1-NKEF, the NKEF expression was dose- and time-dependent, and the highest NKEF expression was detected with 2 µg of plasmid at 6 days post-transfection (Figure 4a). Moreover, we evaluated the activation of IFN1-related genes in RBCs overexpressing NKEF (2 µg of pmTFP1-NKEF at 3 days post-transfection) and we found an increment in *irf3*, *mx*, and *isg15* expression correlated with *nkef* gene expression (Figure 4b and Supplementary Table S1).

### 3.4. Evaluation of NKEF Overexpression on VHSV Infectivity in EPC Cells

**Figure 5:**
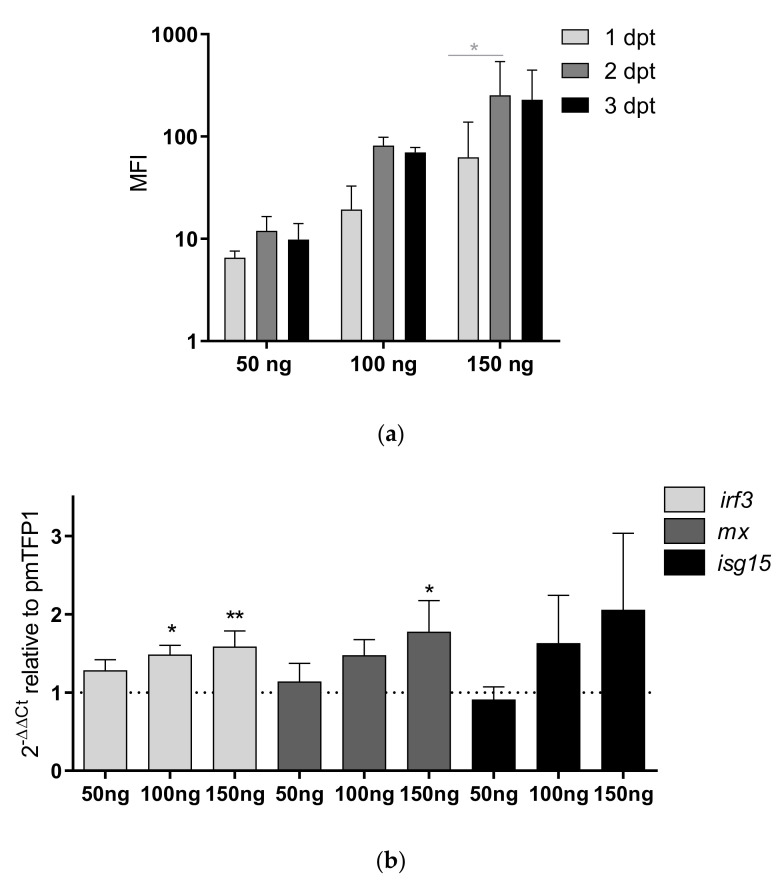
Time course of transfected EPC cells with pmTFP1-NKEF. EPC cells were electroporated with the plasmid pmTFP1-NKEF at different concentrations (50 ng, 100 ng, and 150 ng). NKEF expression was monitored at various time points. (**a**) Evaluation of NKEF protein levels using flow cytometry via the fluorescence emitted by the NKEF-bound TFP1 protein. Data represent mean fluorescent intensity (MFI)(mean ± SD of two experiments). h indicates hours. A two-way ANOVA and Tukey’s multiple comparisons test were performed for statistical analysis, between plasmid concentrations and times post-transfection (gray lines and asterisks). * indicates a *p*-value of <0.05. (**b**) Analysis of the IFN1-related genes (*irf3, mx, isg15*) modulation in EPC cells transfected with the plasmid pmTFP1-NKEF at 48 h. Gene expression was evaluated by RT-qPCR, normalized to pmTFP1 transfected cells (control, dashed line). C*arp-ef1α* was used as endogenous control gene. Data represent the mean ± SD (n = 2 experiments). Bars represent the expression of *irf3* (light grey), *mx* (dark grey) and *isg15* (black). The Kruskal–Wallis test and Dunn’s multiple comparison post hoc tests were performed and compared with the control. * and ** indicates a *p*-value of <0.05 and 0.01 respectively.

To evaluate whether the overexpression of NKEF prevents VHSV infectivity, we performed a transfection assay with pmTFP1–NKEF in EPC cells, since these cells are highly susceptible to VHSV infection [37] and to gene transfection [35]. Firstly, NKEF expression in EPC cells transfected with pmTFP1-NKEF was evaluated at different concentrations (50, 100, and 150 ng) and time points (24, 48, and 72 h post-transfection) by flow cytometry. In EPC cells the NKEF expression was dose- and time-dependent, and the highest NKEF expression was detected with 150 ng of plasmid at 48 h post-transfection (Figure 5a). Therefore, we chose this condition to evaluate the effects of NKEF overexpression on VHSV infectivity in EPC cells. In addition, we evaluated the stimulation of IFN1-related genes such as *mx*, *irf3* and *isg15* in EPC cells overexpressing NKEF and we found an upregulation of the three IFN1-related genes tested, dose dependent on pmTFP1-NKEF transfected concentration (Figure 5b).

**Figure 6:**
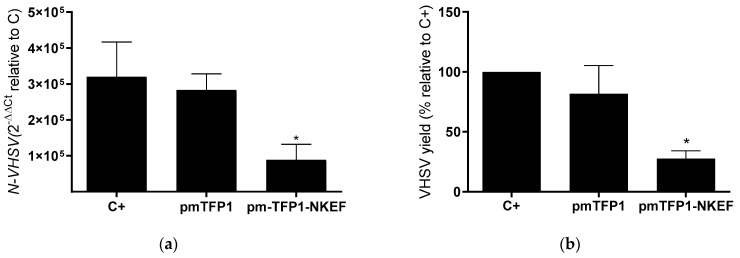
VHSV infectivity in EPC cells transfected with pmTFP1-NKEF. EPC cells were transfected with pmTFP1-NKEF. At 48 h post-transfection, cells were exposed to VHSV MOI 0.1 for 24 h at 14 °C. Cells transfected with empty plasmid (pmTFP1) were used as a negative control. (**a**) VHSV replication in transfected EPC cells analyzed by RT-qPCR of the N-VHSV gene. N-VHSV gene expression was normalized to uninfected cells. C*arp-ef1α* was used as a reference gene. C+ represents VHSV-infected control and C means uninfected cells. Data represent the mean ± SD of 2 experiments. The Kruskal–Wallis test and Dunn’s multiple comparison post hoc test were performed for statistical analysis. * indicates a *p*-value < 0.05. (**b**) Evaluation of VHSV titer in the supernatant of EPC cells transfected with pmTFP1-NKEF. VHSV yield represented as percentage of virus FFU/mL relative to positive control (untransfected and infected cells, C+). Data represent the mean ± SD of two experiments. The Kruskal–Wallis test and Dunn’s multiple comparison post hoc test were performed for statistical analysis. * indicates a *p*-value < 0.05.

At 48 h post-transfection, cells were infected with VHSV at MOI 0.1 for 24 h at 14 °C. The results showed a statistically significant decrease in VHSV replication by means of RT-qPCR (Figure 6a). VHSV yield was also significantly decreased (73%) in the supernatant of EPC cells transfected with pmTFP1–NKEF (Figure 6b).

## 4. Discussion

The aquaculture industry has experienced constant growth since 1950 [38]. According to the 2020 APROMAR report, fish production was 17% higher in aquaculture than fisheries. Thus, aquaculture is a major source for the global food supply. However, economic and social aspects of aquaculture are impacted by viral infections [39] and thus it becomes a need to explore antiviral solutions to this problem.

In spite of the fact that there has been a spectacular development of the knowledge of fish immunology in the recent decades, the fish immune system is not as fully understood as the immune system of mammals. One of the main differences between fish and mammal immune systems lies in the RBCs. Nucleated RBCs characteristic of fish, amphibians, reptiles, and birds are multifunctional cells. In addition to participating in gas exchange and transport, these cells respond to several pathogens, including viruses [40,41]. Our laboratory previously demonstrated that rainbow trout RBCs exposed to viruses such as VHSV and infectious pancreatic necrosis virus (IPNV) increased the expression of proteins that participate in the antiviral immune response [18,23]. Moreover, many antiviral effectors (e.g., interferon-induced proteins with five tetratricopeptide repeats [*ifit5*], cholesterol 25-hydroxylase [*ch25h*], GTPase, a very large interferon-inducible pseudogene 1 [*gvinp1*], Mx dynamin like GTPase [*Mx*], interferon induced protein 35 [*ifi35*], radical S-adenosyl methionine (SAM), domain-containing protein 2 [*rsad2*, also known as viperin], interferon-induced transmembrane protein 3 [*ifitm3*], tripartite motif [trim] gene family, and sterile alpha motif (SAM) and histidine-aspartate (HD) domain-containing protein 1 [*samhd1*]) were up-regulated in a transcriptomic analysis of RBCs from rainbow trout individuals challenged with VHSV [21]. In addition, IFIT5 was identified in the RBC proteome immunoprecipitated with the N protein of VHSV after exposure to the virus, and its participation in the RBC antiviral immune response was detailed [20]. NKEF was also identified in the RBC proteome immunoprecipitated with the N protein of VHSV. In the current study, we evaluated the expression profile of *nkef* gene in two rainbow trout cell types with different susceptibility to VHSV infection, such as RTG-2 cell line and RBCs. We found that *nkef* gene expression was almost unchanged in RTG-2 infected cells. In contrast, we found that *nkef* gene expression increased in RBCs in response to VHSV exposure at 6 hpe with a recovery to basal levels at 24 hpe onwards. In addition, we found a nonstatistically significant increment at protein level. In a proteomic analysis of RBCs from rainbow trout challenged with VHSV, a downregulation of NKEF protein expression was observed at two days postchallenge (in Supplementary Materials) [21]. However, in this study, increased *nkef* gene expression has been found in spleen, head kidney and RBCs from rainbow trout challenged with VHSV. Similar results have been described in peripheral blood leukocytes and head kidney from VHSV-challenged rainbow trout [14,15], in peripheral blood leukocytes from SVCV-challenged carp [16], and in the brain and head kidney of nodavirus- infected seabream and sea bass [19]. In addition, *nkef* gene expression has been described to be increased in the blood, spleen, and liver of rockfish (*Sebastes schlegelii*) after a stimulation with polyinosinic:polycytidylic acid (poly I:C), a mimetic of viral RNA [42]. It is worth noting that genome duplication in teleost species [43] could influence the diverse *nkef* gene and protein expression profiles found within species. However further studies are needed to corroborate this.

Additionally, *nkef* gene silencing resulted in increased VHSV replication in RBCs and in RTG-2 cells, indicating that NKEF was involved in the antiviral mechanisms of rainbow trout RBCs against VHSV and supporting the antiviral role of this protein. Similar results were found after *ifit5* gene silencing in RBCs [20]. Similarly, knockdown of Prdx1 by siRNA increased hepatitis B virus propagation in human hepatoma Hep38.7-Tet cells [44]. In contrast, knockdown or knockout of Prdx1 expression in mice embryonic fibroblasts inhibited influenza A viral replication, indicating that Prdx1 is essential for influenza A virus replication [45].

Finally, we evaluated the antiviral ability of NKEF against VHSV by its overexpression in EPC cells using a plasmid encoding *nkef* gene (pmTFP1-NKEF). The use of plasmid vectors to overexpress antiviral proteins in fish, such as type I IFNc in Atlantic salmon against ISAV and black carp Mx1 against SVCV and grass carp reovirus, has been previously reported [46,47]. Our results demonstrated that NKEF overexpression reduced VHSV infectivity and virus replication in transfected EPC cells. Similar results have been reported in turbot injected with a plasmid expressing NKEF and subsequently infected with megalocytivirus, where viral loads were significantly reduced [48]. Recombinant NKEF-A and NKEF-B proteins have been reported to inhibit HIV-1 replication in human H9 T-cells [11]. These findings collectively suggest the antiviral potential of NKEF, although the mechanism of the antiviral action is mostly unknown. It was recently reported that human PRDX1 colocalizes and interacts with influenza virus ribonucleoproteins in human cell lines [45]. Notably, we have not observed a clear colocalization between VHSV and NKEF in VHSV-infected EPC cells transfected with pmTFP1–NKEF (data not shown). However, a slight but not conclusively colocalization was observed in RBCs exposed to VHSV at an early time postexposure (Supplementary Figure S2). In addition, NKEF was reported to immunoprecipitate with the N protein of VHSV [20]. Therefore, NKEF may be interacting with VHSV, but further experiments are needed to explore this possibility.

Another potential antiviral mechanism of NKEF could be the modulation of the immune response. We have found a downregulation of IFN1-related genes (*irf3*, *mx*, *isg15*) in RBCs and RTG-2 transfected with siNKEF and exposed to VHSV. In addition, a modulation of IFN1-related genes was found in RBCs and EPC cells overexpressing NKEF. Therefore, these results indicate that NKEF might be implicated in the modulation of the IFN1-related cell response. To our knowledge, this is the first time that NKEF is related to IFN1 pathway modulation as an explication of its role in the antiviral immune response. Solely, NKEF has been documented to interact with IRF3 [49] and ISG15 [50] in protein–protein interaction network studies. Therefore, our results open up new avenues of research around this protein.

## 5. Conclusions

In summary, previously we identified NKEF in the immunoprecipitated proteome of rainbow trout red blood cells (RBCs) exposed to viral hemorrhagic septicemia virus (VHSV), indicating the possible implication of this protein with the viral infection. Therefore, in this article we have evaluated the role of this protein as antiviral molecule in rainbow trout. We demonstrated that NKEF is involved in the antiviral mechanisms of rainbow trout against VHSV, through protein silencing and overexpression in different fish cells types. In addition, we have demonstrated that NKEF might be implicated in the modulation of the IFN1-related cell response. In conclusion, we determined the antiviral effect of this protein, providing evidence that NKEF may be a potential target for new therapeutic strategies against viral infections.

## Figures and Tables

**Table 1:** Sequences of rainbow trout *nkef*-specific siRNA.

Name	siRNA Design Sequence (5′–3′)	Start on Target
siNKEF-1 sense	CGUAUAGCUUGGAGAUGUUdTdT	840
siNKEF-1 antisense	AACAUCUCCAAGCUAUACGdTdT	840
siNKEF-2 sense	CAAACUAUGAAGAUUAUAUdTdT	818
siNKEF-2 antisense	AUAUAAUCUUCAUAGUUUGdTdT	818

**Table 2:** Sequences of primers used for RT-PCR.

Gene	Forward Primer (5′–3′)	Reverse Primer (5′–3′)	Annealing, Ta (°C)	Reference or Accession Number
*nkef*	TCCAAGCAGCAGTAAGACGA	CATGAGATAAGGGGATGCTGA	60	AF250194.1
*gapdh*	ATGTCAGACCTCTGTGTTGG	TCCTCGATGCCGAAGTTGTCG	52	[28]
*Β-actin*	AAGTGTGACGTGGACATCCG	CAGAGCTGAAGTGGTAGTCGG	60	NM_001124235.1

**Table 3:** Sequences of primers and probes for RT-qPCR.

Gene	Forward Primer (5′–3′)	Reverse Primer (5′–3′)	Probe (5′–3′)	References or Accession Number
*Om-nkef*	CGCTGGACTTCACCTTTGTGT	ACCTCACAACCGATCTTCCTAAAC	-	[18]
*N-VHSV*	GACTCAACGGGACAGGAATGA	GGGCAATGCCCAAGTTGTT	TGGGTTGTTCACCCAGGCCGC	[27]
*Om-ef1α*	ACCCTCCTCTTGGTCGTTTC	TGATGACACCAACAGCAACA	GCTGTGCGTGACATGAGGCA	[29]
*Om-mx*	TGAAGCCCAGGATGAAATGG	TGGCAGGTCGATGAGTGTGA	ACCTCATCAGCCTAGAGATTGGCTCCCC	[30]
*Om-isg15*	GTTAGGCGTCAATGGGAACAA	GGCCATAGTCGCTCAAAGTTTT	-	XM_036979883.1
*Om-irf3*	AACAAGGCATGCAGGGTTCTAAAT	ACGTGTGCAATCAGTACCAGCA	-	[31]
*Carp-ef1α*	CTGGAGGCCAGCTCAAACT	CATTTCCCTCCTTACGCTCAAC	-	AY643400
*Carp-mx*	GGA GAA GAG GTT AAA TGT GGA TCA G	TGA CCG AAT CAA GAA GTC ATT CC	-	[32]
*Carp-isg15*	TAATGCCACAGTCGGTGAA	AGGTCCAGTGTTAGTGATGAGC		[33]
*Carp-irf3*	GTTTAGAGGGACAATTAACTGGACTA	GAGGGTCCACTCTTTGAAAATG		[33]

## Data Availability

The raw data of this article will be made available by the authors without reservation.

## Supplementary Materials

The following are available online at https://www.mdpi.com/article/10.3390/biology10101045/s1, Table S1: IFN1-related genes *irf3*, *mx*, and *isg15* modulation in RBCs transfected with the plasmid pmTFP1-NKEF. Gene expression was evaluated by RT-qPCR. Data represent pmTFP1-NKEF transfected RBCs (with 2 µg of plasmid and at 3 dpt). Gene expression was normalized to pmTFP1 transfected RBCs. The endogenous gene control used was *om-ef1α*, n = 3 individuals, Figure S1. NKEF protein expression determined by Western blot in RTG-2 electroporated with siNKEF sequences at 24 and 48 h. (a) Representative image of Western blot of NKEF protein levels after NKEF gene silencing in RTG-2 cells after 24 and 48 h using anti-NKEF antibody. (b) Representative image of α-actin used as endogenous protein control detected using anti-α-actin antibody. C means non electroporated control; C- means control mock electroporated; siGFP means cells electroporated with siGFP at 48 h; h means hours, Figure S2: PLA quantification between NKEF and GVHSV. PLA from RBCs exposed to VHSV (MOI 1 for 6 h), were performed using specific antibodies against NKEF and protein GVHSV. The percentage of NKEF-GVHSV positive cells in the PLA was represented in the graph. Data represent the mean ± SD, n = 2. A Mann-Whitney test was performed for statistical analysis.
