# Supplementary figures and images for: Antiviral Function of NKEF against VHSV in Rainbow Trout
Source: Biology (Basel). 2021 Oct 15;10(10):1045. doi: 10.3390/biology10101045 (PMC8533630; doi:10.3390/biology10101045)

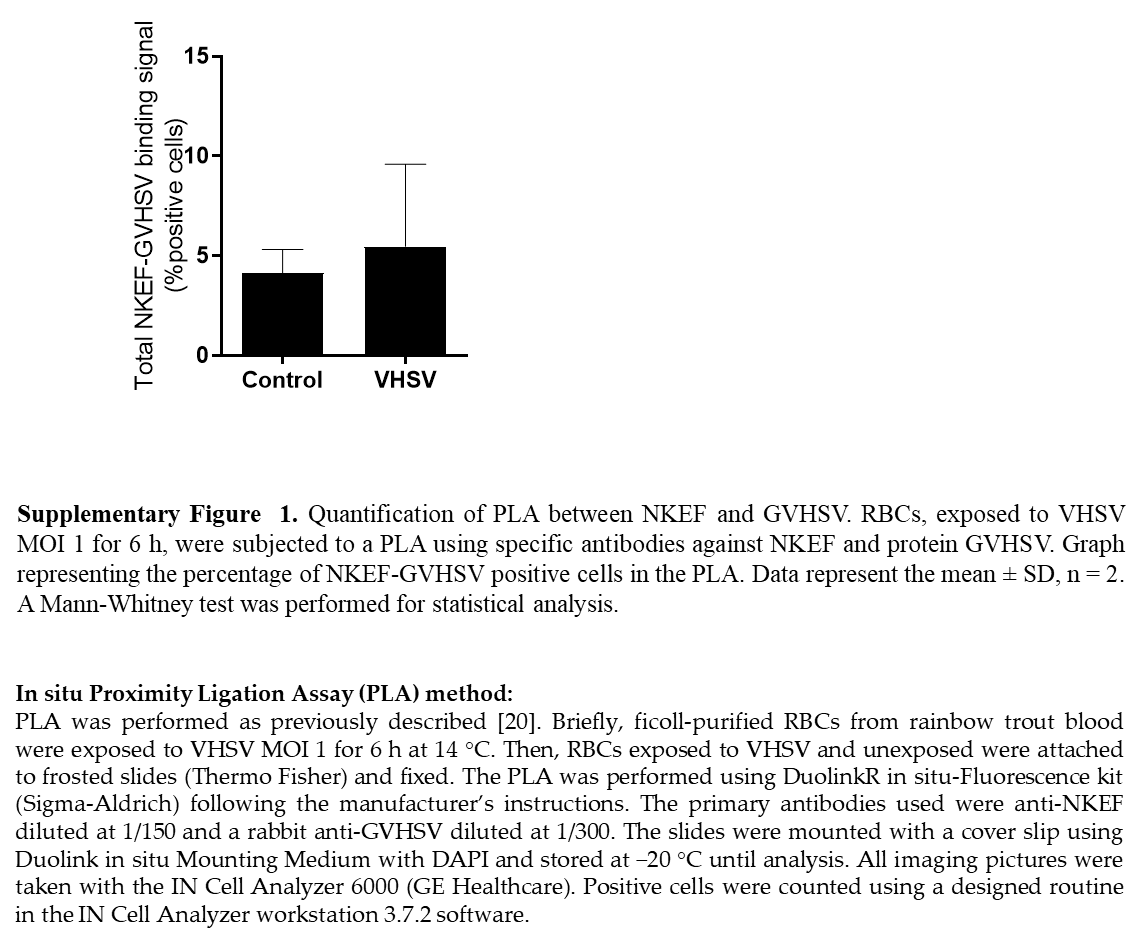

Supplement: Supplementary file 1 [file biology-10-01045-s001.zip › Supplementary FigureS1_revision1.tif]

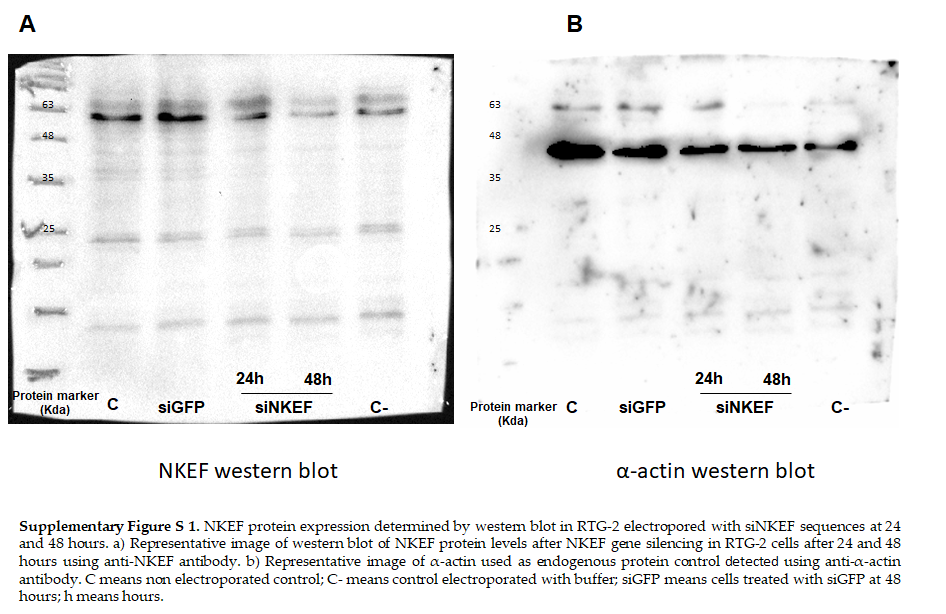

Supplement: Supplementary file 1 [file biology-10-01045-s001.zip › Supplementary FigureS2_revision1.tif]
